# Effects of weights applied to the apex of a bag-valve-mask and pinch strength on tidal volume: a prospective simulation study

**DOI:** 10.1038/s41598-024-54098-6

**Published:** 2024-02-13

**Authors:** Dong Choon Uhm, A Jung Kim, Bong Yeun Koh, Kui Ja Lee

**Affiliations:** 1 Department of Paramedicine, Daejeon University, Daejeon, 34520 Republic of Korea; 2 Department of Paramedicine, Kyungil University, Gyeongsan, 38428 Republic of Korea; 3 Department of Nursing, Dongnam Health University, Suwon, 16328 Republic of Korea; 4 Department of Paramedicine, Kyoundong University, 815, Gyeonhwon-ro, Munmak-eup, Wonju-si, Gangwon-do 26495 Republic of Korea

**Keywords:** Bag-valve-mask, Pinch strength, Tidal volume, Ventilation, Health care, Medical research

## Abstract

A bag-valve-mask (BVM) is a first aid tool that can easily and quickly provide positive-pressure ventilation in patients with breathing difficulties. The most important aspect of BVM bagging is how closely the mask adheres to the patient’s face when the E–C technique is used. In particular, the greater the adhesion force at the apex of the mask, the greater the tidal volume. The purpose of this study was to investigate the effect of various weights applied to the mask’s apex and the pinch strength needed to perform the E–C technique, on tidal volume. In this prospective simulation study, quasi-experimental and equivalent time-series designs were used. A total of 72 undergraduate paramedic student from three universities were recruited using convenience sampling. The tidal volumes according to the weights (0 g, 100 g, 200 g, 300 g) applied to the apical area of the mask, handgrip strength, and pinch strength (tip pinch strength, key pinch strength, and tripod pinch strength) were measured. A linear mixed model analysis was performed. Linear mixed model analyses showed that tidal volume was significantly higher at 200 g (B = 43.38, *p* = 0.022) and 300 g (B = 38.74, *p* = 0.017) than at 0 g. Tripod pinch strength (B = 12.88, *p* = 0.007) had a significant effect on mask adhesion for effective BVM ventilation. Adding weight to the apical area of the mask can help maintain the E–C technique and enable effective ventilation. Future studies are required to develop specific strategies to improve the ventilation skills, which can be an important first-aid activity.

Positive-pressure ventilation can be implemented by manual methods, such as bag-valve-mask (BVM) bagging, and automatic methods, such as the use of ventilators. In emergency situations including those that arise during epidemics of respiratory infectious diseases, such as coronavirus disease 2019 (COVID-19), the rescuer’s first action is to provide positive-pressure ventilation using a BVM^1–3^. The rescuer places the mask around the patient’s nose and mouth with one hand for BVM bagging, closes the gap between the mask and the face using the E–C technique with their thumb and index finger, and opens the airway. With the other hand, the self-inflating bag of the BVM is squeezed so that the ventilation is 500–600 mL.

BVM bagging has the advantage of providing the fastest and easiest positive-pressure ventilation anytime and anywhere, without needing special mechanical devices. However, the volume of BVM ventilation differs according to the rescuer’s experience level^4–6^. Previous studies have reported that BVM bagging results in greater air leakage and lower oxygen saturation than mechanical ventilation^7–9^.

One of the most important aspects of BVM bagging is the complete sealing of the mask to the patient’s face, as mask leakage will result in reduced tidal volume delivered to the patient’s lungs^10^. Previous studies have investigated the sealing of masks to provide appropriate ventilation during BVM bagging^11,12^. To provide appropriate ventilation without side effects, the mask in BVM was improved so that the entire mask could be controlled with one hand by making a power grip on the mask apex to seal the mask to the face well^13^, and the BVM was modified by adding a supplemental internal handle^12^ and external handle^14^ or using the pediatric BVM for adults^15^. In addition, a study measuring the 4-spot adhesion strength (sealing forces measured at the bottom, left side, right side and apex of the mask) between a mask and a manikin’s face using the E–C technique demonstrated that the tidal volume (Vt) increases when the apex sealing force during BVM ventilation increases^16^. However, it is difficult to apply more force to the apex of the mask when using a one-handed E–C technique for ventilating the patient; therefore, it is necessary to consider shifting the center of gravity to the apex of the mask.

Although previous studies have reported that the factors affecting effective ventilation were sex, height, weight, C length in the E–C technique, handgrip power, hand width, hand length^11,16,17^, it is difficult to confirm which factors affect BVM ventilation, especially because few follow-up studies use the same variables. Nevertheless, the rescuer’s hand function is important for appropriate ventilation because BVM bagging with the E–C technique requires the hands^11,18^. In addition, the fingers play an important role in maintaining the E–C technique for effective ventilation^6^. In order to prevent air leakage during BVM bagging, the rescuer can adhere the mask to the patient's face in an EC shape using the palms and fingers as a hand function. One of the important hand functions is hand strength, and which can measure various gripping forces such as grip strength, tip pinch, key pinch, and tripod pinch^19^. Pinch strength is measured using the thumb, index finger, and middle finger, which indicates the finger strength. However, few studies have measured the effects of ventilation using the same variables as pinch strength.

Based on the results of our previous studies^16,20^, we further investigated the effect of ventilation by weighing the apical area for mask adhesion and measuring the pinch strength required to maintain the E–C technique. Thus, this study aims to identify the varying effects on Vt according to four weights (0 g, 100 g, 200 g, 300 g) applied to the apical area of the mask and pinch strength.

## Methods

### Study design

This prospective simulation study was conducted using quasi-experimental and equivalent time series designs.

### Sample size and setting

To achieve a reasonable sample size, we performed a power analysis using G*Power 3.1.9.4^21^ for linear mixed model (LMM) analysis. The sample size for computation of a test power (1 − *β*) of 0.80 with seven predictors was 68, with an effect size of 0.15 and alpha of 0.05. Considering the dropout rate, 75 students were recruited to achieve a reasonable sample size. During the study, three students withdrew owing to personal problems. In total, 72 students were included in the analysis.

Using convenience sampling, participants were recruited from three universities in three large cities in the Republic of Korea. The inclusion criteria were undergraduate paramedic students aged 18 years or older who had completed a basic life support provider course, according to the 2020 American Heart Association guidelines^3^. The participants were held the mask in their left hand. This study was conducted during the COVID-19 pandemic. Therefore, students who contracted COVID-19 and hence needed to be quarantined were excluded.

The principal researcher contacted the directors of the paramedicine departments of the three universities to obtain permission for recruitment. Data were collected between November 14, 2022 and February 28, 2023.

### Ethical considerations

This study was conducted in accordance with the principles of the Declaration of Helsinki and was approved by the institutional review board of Kyungdong University (No. 1041455-202210-HR-015-01). Participation was voluntary and anonymous. Before conducting the study, we received informed consent from all participants, who were informed that consent could be withdrawn at any time during the study without any consequences.

### Measurements and procedures

**Figure 1:**
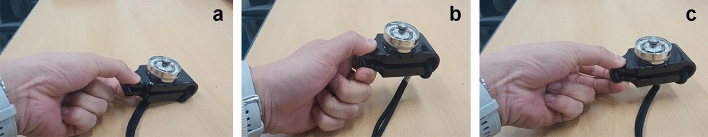
Pinch strength position. (**a**) Tip pinch, (**b**) key pinch, and (**c**) tripod pinch.

General characteristics (such as sex, grade, height, and weight), and major variables included handgrip strength, three pinch strengths (tip, key, and tripod), and Vt were measured in this study. First, general characteristics were measured using a self-reported questionnaire. The handgrip strength (kg) and three pinch strengths (kg) of the objective variables were then measured using an electronic hand dynamometer (Lavisen KS-301, Lavisen Co. Ltd., Namyangju, Republic of Korea) and Jamar Pinch Gauge (PG 60, Sammons Preston, Bolingbrook, IL, USA), respectively. Handgrip strength (kg) and pinch strength (kg) were measured twice with the left hand, and the average values were obtained. The pinch strengths included tip, key, and tripod pinch strengths (Fig. 1). Each participant was measured pinch strengths in three types of finger shapes (postures) as shown in Fig. 1. Tip pinch strength is the force between the base of the thumb and the tip of the index finger, and key pinch strength is the force between the anterior of the thumb and the inner side of the index finger. The tripod pinch strength is the force between the anterior of the thumb and the anterior surfaces of the index and middle fingers^19^.

Vt (mL) was measured via BVM bagging (Adult Laerdal Silicone Resuscitator, Laerdal Medical Corporation, Stavanger, Norway). In this study, Brayden Pro manikin (Innosonian, Inc., Seoul, Republic of Korea), which has a built-in software that can automatically measure Vt, was used. Based on previous studies, the Brayden Pro manikin was placed at the same height as the middle of the femur of the participants^22^.

**Figure 2:**
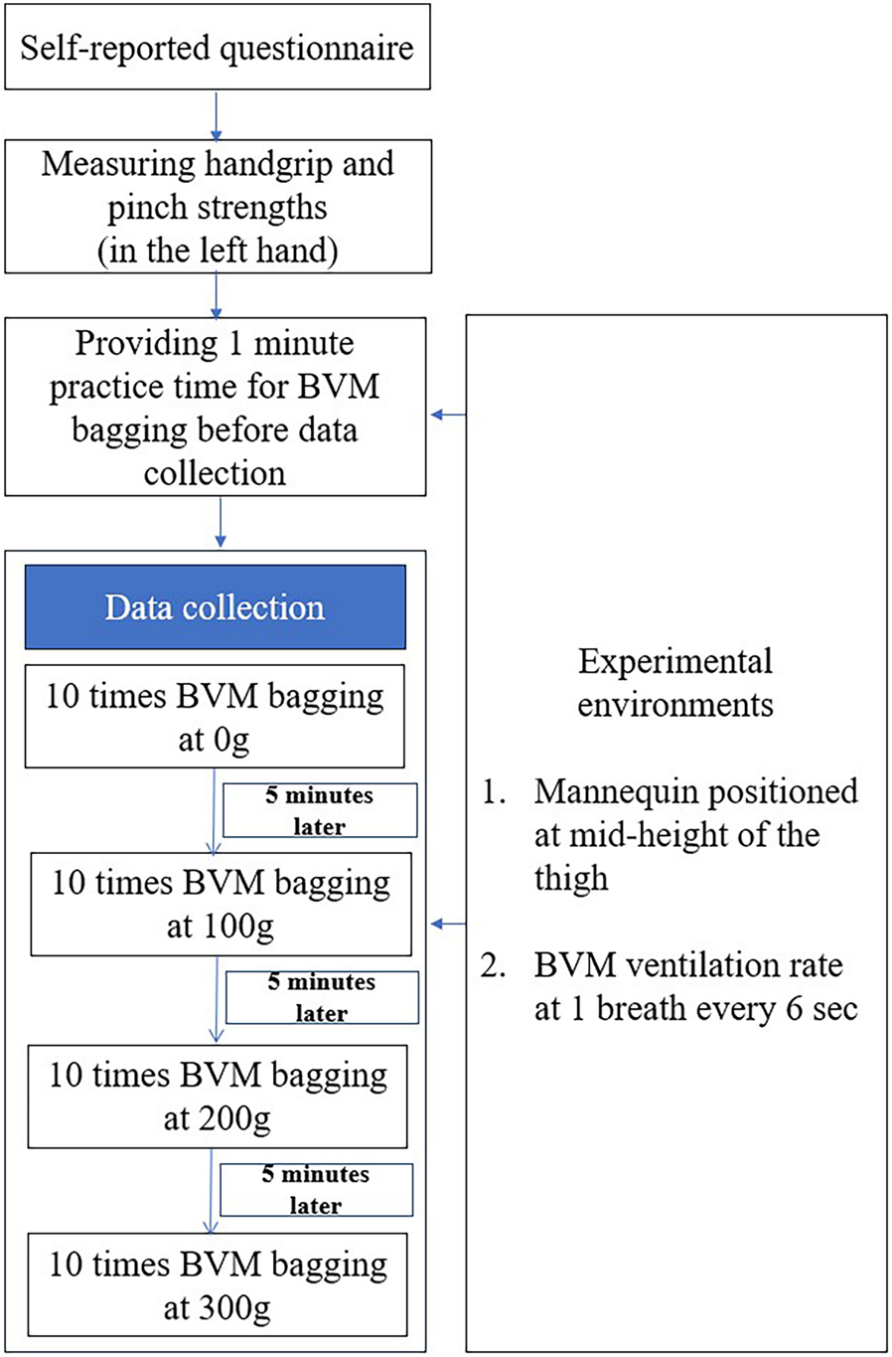
Flowchart of the study design.

Machine usage was demonstrated and explained data collection. All participants were given the opportunity to practice BVM bagging for 1 min while maintaining the E–C technique with their left hand and holding the self-inflating BVM bag with their right hand. In addition, the participants were given a 5-min break after measuring each variable to minimize their difficulty. It took approximately 35–40 min to measure all the variables for each participant (Fig. 2).

**Figure 3:**
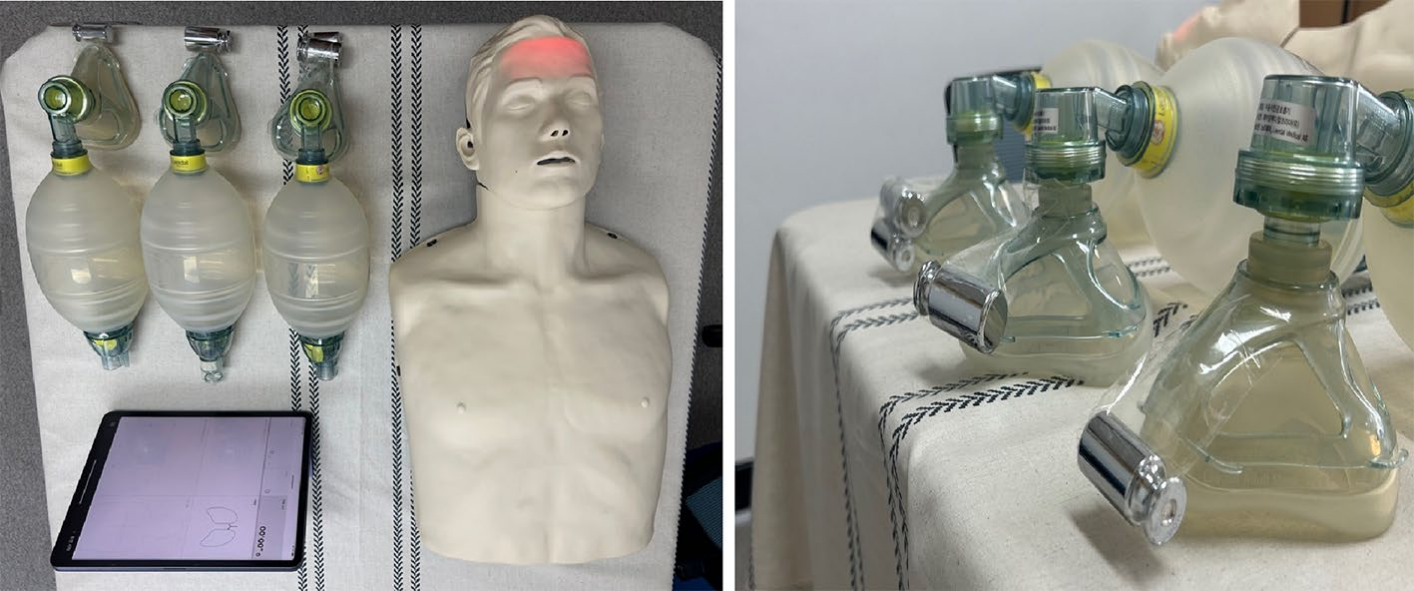
Experimental environment.

Mean Vt was measured by BVM ventilation for 1 min for each pendulum (0 g, 100 g, 200 g, 300 g, respectively). The measured Vt was calculated 10 times for each weight placed on the mask, and the average value of Vt (mL) measured according to each weight was calculated. A metronome (Metronome Beats: BPM Counter, App Store, Stonekick Limited, London, United Kingdom) was used to accurately provide ventilation rates to measure the Vt (mL). It was set to 60 beats per minute, allowing participants to administer one ventilation every 6 s (Fig. 3).

### Statistical analysis

Data were analyzed using SPSS Statistics for Windows, Version 27.0 (IBM SPSS Inc., Chicago, IL, USA). To confirm the normal distribution of the data, kurtosis (< 10) and skewness (< 3) were calculated. Statistical analyses included descriptive statistics, t-tests, Pearson’s correlation analysis, and LMM analyses. To obtain accurate Vt in the correlation analysis, parameters such as the four weights, height, tripod pinch strength, and handgrip strength, which were found to display positive correlations, were treated as the control variables and included in the LMM analysis. For the independent variables, dummy (sex and grade) codes were created to run the LMM analysis.

## Results

### General characteristics and variation in tidal volume

**Figure 4:**
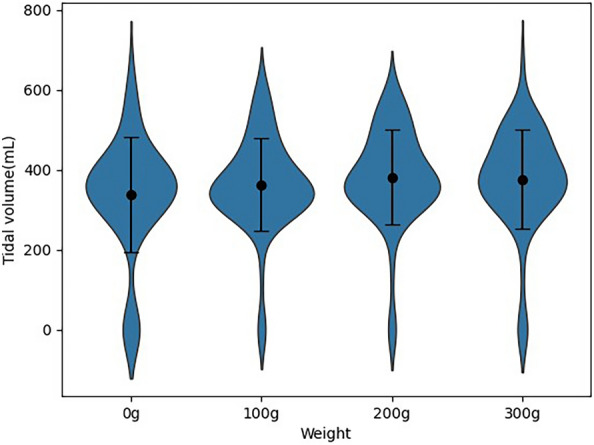
Violin plots and mean/standard deviation (black circle, vertical bar) for the variation in tidal volume with pendulum weight.

Of the 72 participants, 51 (70.8%) were female. The general characteristics of the participants are listed in Table 1. Among the three types of average pinch strengths, the key pinch strength was the highest at 7.55 (± 2.64) kg followed by the tripod pinch strength (6.71 ± 2.46 kg) and tip pinch strength (6.54 ± 2.19 kg; Table 1). Among the weights applied to the apical area of the mask, 200 g resulted in the highest Vt at 381.18 (± 118.65) mL followed by 300 g, which resulted in a Vt of 376.54 (± 124.93) mL (Fig. 4). The Vt according to weight by sex was the highest with 300 g for males and 200 g for females (419.52 ± 74.90 mL vs. 376.47 ± 119.23 mL; Table 2). However, the Vt for each weight according to sex was not statistically significant. The Vt according to the weight by grade was the highest at 200 g (395.58 ± 117.31 mL) for the 3rd grade and 300 g (370.10 ± 111.38 mL) for the 4th grade (Table 2). The Vt according to grade was also not statistically significant.

**Table 1:** General characteristics.

Variables	n = 72
Sex	
Male	21 (29.2)
Female	51 (70.8)
Grade	
3rd	43 (59.7)
4th	29 (40.3)
Height (cm)	166.33 ± 7.87
Weight (kg)	63.76 ± 13.78
Handgrip strength (kg)	32.74 ± 10.65
Pinch strength (kg)	
Tip	6.54 ± 2.19
Key	7.55 ± 2.64
Tripod	6.71 ± 2.46
Mean tidal volume (mL)	
0 g	337.81 ± 143.39
100 g	363.17 ± 115.77
200 g	381.18 ± 118.65
300 g	376.54 ± 124.93

Data are expressed as mean ± standard deviation or n (%), as appropriate.

**Table 2:** Comparison of tidal volume by sex and grade.

Tidal volume (mL)	Sex (n = 72)		t	*p*	Grade (n = 72)		t	*p*
	Male (n = 21)	Female (n = 51)			3^rd^ (n = 43)	4^th^ (n = 29)		
0 g	356.48 ± 148.48	330.12 ± 142.02	0.71	0.482	347.40 ± 131.06	323.59 ± 161.31	0.69	0.493
100 g	377.48 ± 78.04	357.27 ± 128.35	0.67	0.505	359.91 ± 126.04	368.00 ± 100.57	− 0.29	0.773
200 g	392.62 ± 119.33	376.47 ± 119.23	0.52	0.603	395.58 ± 117.31	359.83 ± 119.43	1.26	0.212
300 g	419.52 ± 74.90	358.84 ± 137.19	1.91	0.061	380.88 ± 134.40	370.10 ± 111.38	0.36	0.722

Data are expressed as mean ± standard deviation.

## Correlation between tidal volume and general characteristics

Table 3 presents the results of the correlation analyses. Among the three types of pinch strengths, the tripod pinch strength was positively correlated with Vt depending on the weight (0 g: *p* = 0.002; 200 g: *p* = 0.032; and 300 g: *p* = 0.003). Additionally, the handgrip strength was positively correlated with Vt at 300 g (*p* = 0.016). In addition, the correlation between height and Vt depending on the weight 0 g (*p* = 0.031) and 300 g (*p* = 0.005) was positive (Table 3).

**Table 3:** Correlation analysis of tidal volume according to general characteristics.

Variables	Mean tidal volume (mL)			
	0 g	100 g	200 g	300 g
	r (*p*)	r (*p*)	r (*p*)	r (*p*)
Sex^1)^	− 0.08 (0.482)	− 0.08 (0.505)	− 0.06 (0.603)	− 0.22 (0.061)
Grade^2)^	− 0.08 (0.493)	0.03 (0.773)	− 0.15 (0.212)	− 0.04 (0.722)
Height	0.25 (0.031)	0.15 (0.195)	0.18 (0.134)	0.32 (0.005)
Weight	0.18 (0.138)	0.20 (0.086)	0.12 (0.322)	0.14 (0.232)
Pinch strength				
Tip	0.05 (0.683)	0.22 (0.066)	0.18 (0.130)	0.13 (0.294)
Key	0.14 (0.225)	0.05 (0.681)	0.03 (0.816)	0.11 (0.370)
Tripod	0.36 (0.002)	0.18 (0.125)	0.25 (0.032)	0.35 (0.003)
Handgrip strength	0.17 (0.156)	0.15 (0.212)	0.16 (0.183)	0.28 (0.016)

Date are expressed as Pearson’s correlation coefficient.

Significant values are in [underline].

### LMM analysis among variables

The results of the LMM analysis are presented in Table 4. Among the control variables (including height, tripod pinch strength, and handgrip strength), tripod pinch strength had a significant effect only on Vt (*p* = 0.007). In the analysis of the weight applied to the apical area of the mask, 0 g was used as the reference variable. The LMM analysis revealed that the Vt was significantly higher at 200 g (*p* = 0.022) and 300 g (*p* = 0.017) than at 0 g (Table 4).

**Table 4:** Linear mixed model (LMM) analysis.

Variables	Estimate	SE	t	*p*	95% CI	
					LLCI	ULCI
(Constant)	− 122.84	242.55	− 0.51	0.614	− 605.02	359.34
Pendulums						
0 g	(Reference)					
100 g	25.36	19.74	1.28	0.2	− 13.5	64.22
200 g	43.38	18.85	2.3	0.022*	6.26	80.49
300 g	38.74	16.16	2.4	0.017*	6.86	70.61
Height	2.38	1.61	1.48	0.143	− 0.82	5.59
Tripod pinch strength	12.88	4.62	2.79	0.007**	3.69	22.07
Handgrip strength	− 0.68	1.27	− 0.54	0.594	− 3.22	1.85

*SE* standard error, *CI* confidence interval, *LLCI* low-limit confidence interval, *ULCI* upper-limit confidence interval.

*p < 0.05, **p < 0.01.

## Discussion

This study was conducted to identify the effects of the application of four different weights to the apical area of the mask and pinch strengths, which are required to maintain E–C technique, on Vt during BVM bagging. The mean Vt by sex and grade in this study were 356 mL and 330 mL, respectively; the Vt at the 3rd grade was 347 mL and that at the 4th grade was 323 mL. These results are not only lower than the results of previous studies^16,20^, but also less than the volume (6–7 mL/kg) recommended by the American Heart Association. Regarding the variation in Vt according to weight, although the mean Vt increased when the weight increased from 100 to 200 g, the Vt at 300 g was 5 mL lower than that at 200 g. This result was attributed to the difference in the number of pendulums applied to the apical area of the mask during BVM bagging. To apply 100 g and 200 g to the mask, one 100 g and one 200 g pendulum were used respectively. However, to apply 300 g to the mask, 100 g and 200 g pendulums were attached and used. The use of two attached pendulums rather than one pendulum may have reduced the space available for maintaining the E–C technique. However, follow-up studies are required to accurately identify the causes of these results. Nevertheless, Vt increased as the weight increased compared with the Vt obtained with the traditional method (0 g at the apex of the mask).

In the correlation analysis, height was positively correlated with Vt, consistent with a previous study^11^. Among the four weights, handgrip strength was positively correlated with Vt at 300 g. Although this result is similar to the results of previous studies^5,12^, it is difficult to compare them because the previous studies did not use the same research method used in this study. A correlation was observed between Vt and the tripod pinch strength when 0 g, 200 g, and 300 g pendulums were applied to the apical area of the mask.

In the LMM analysis, Vt increased when the weight applied to the apical area of the mask was increased compared with the Vt obtained with traditional BVM bagging (weight = 0 g). Among the four weights, the average Vt increased significantly by 43 mL and 39 mL when 200 g and 300 g, respectively, were applied. Although the average Vt increased by 25 mL even at 100 g weight, this increase was not statistically significant. A previous study also reported that the higher the apex sealing force of the mask, the higher the Vt^16^. Based on these results, it was estimated that when the weight applied on the apex of the mask increases, the sealing force of the apical area increases.

In addition, Vt increased by 12.9 mL per 100 g when applied to the apical area of the mask at tripod pinch strength. Tripod pinch strength refers to the force between the anterior of the thumb and the anterior surface of the index and middle fingers^19^. In the E–C technique, the thumb and index finger used to measure tripod pinch strength form a “C” shape, and the middle finger forms an “E” shape. Therefore, the execution of the E–C technique can be affected by the tripod pinch strength. In the tripod pinch strength test, there was a difference in Vt according to the weights applied to the apical area of the mask. Based on this result, more strength can be applied to the middle finger used for “C” shape and the finger maintaining the “E” shape. Maintaining the “E” shape when performing the E–C technique is essential for keeping the airway open during BVM bagging^1,5,10,20^. Thus, the pinch strength that affects the E–C technique during BVM bagging is the tripod pinch strength and applying a 200 g weight to the apex of the mask is considered to be a method for improving ventilation by increasing adhesion through the EC technique. However, previous studies^19,23,24^ have reported that data such as arm circumference, hand shape, and hand size should be included to accurately measure pinch strength, as well as the occupation, impetus, and disease history of the patient. To accurately understand the effect of pinch strength on BVM bagging, further studies that include parameters affecting the pinch strength are needed.

This study is significant in three respects. This is the first simulation study to identify the effect of various weights applied to the apex of the mask on BVM ventilation. Second, this study suggests a new method for supplying Vt through a modified E–C technique that effectively seals the mask for optimal BVM ventilation. Finally, the results of this study are necessary for the development of BVM devices.

This study has several limitations. First, the results of this study cannot be said to produce the same results in humans as this was a quasi-experimental study that used a manikin without variables such as airway opening angle, peak pressure, inhalation time, mask sealing force, and air leakage. Second, the results of this study may not be generalizable because the participants were recruited via convenience sampling. Third, in this study, the weights applied to the BVM could not be created using the blind method for the participants. Consequently, it cannot be ruled out that this setup potentially affected the force and posture needed to properly seal the BVM mask, as well as the tidal volume (Vt). Additionally, the weight of the plumb bob might have caused variations in the force applied to seal the BVM mask, which is another factor that cannot be disregarded. Fourth, the participants of this study were only those who hold the mask with the left hand since the previous study^16^ was reported that 92.1% of participants held the mask in their left hand. Therefore, we cannot sure that the same effect occurs in the opposite situation. Finally, the outcomes may depend on the experimental environment such as the type of manikin and BVM device.

## Conclusions

This study assessed the effect of various weights (0 g, 100 g, 200 g, and 300 g) applied to the apex of the mask and the pinch strengths required to maintain the E–C technique during BVM bagging. Among the weights, Vt increased the most during BVM bagging with 200 g and 300 g pendulums, and it was greater with a 200 g pendulum than with a 300 g pendulum. Of the three types of pinch strengths, tripod pinch strength affected Vt the most. The tripod pinch strength is required to maintain “C” shape and “E” shape in the E–C technique. It can be inferred that the weight applied to the apex of the mask provides more strength to the 2nd and 3rd fingers, which affects the tripod pinch strength. Based on these results, it is necessary to train paramedics to apply a weight to the apex of the BVM for optimal BVM ventilation in clinical practice.

## Data Availability

The datasets used and/or analysed during the current study are available from the corresponding author on reasonable request.

## Abbreviations

BVM: Bag-valve-mask
COVID-19: Coronavirus disease 2019
LMM: Linear mixed model
Vt: Tidal volume
